# Current Development in Decolorization of Synthetic Dyes by Immobilized Laccases

**DOI:** 10.3389/fmicb.2020.572309

**Published:** 2020-09-30

**Authors:** Sherine Ahmed Gamal Zakaria Morsy, Asilah Ahmad Tajudin, Mohd. Shukuri Mohamad Ali, Fairolniza Mohd. Shariff

**Affiliations:** ^1^Enzyme and Microbial Technology Research Centre, Faculty of Biotechnology and Biomolecular Sciences, Universiti Putra Malaysia, UPM Serdang, Selangor, Malaysia; ^2^Department of Microbiology, Faculty of Biotechnology and Biomolecular Sciences, Universiti Putra Malaysia, UPM Serdang, Selangor, Malaysia; ^3^Department of Biochemistry, Faculty of Biotechnology and Biomolecular Sciences, Universiti Putra Malaysia, UPM Serdang, Selangor, Malaysia

**Keywords:** immobilization, laccase, oxidoreductase, textile dye wastewater, decolorization, detoxification, biodegradation

## Abstract

The world today is in a quest for new means of environmental remediation as the methods currently used are not sufficient to halt the damage. Mostly, a global direction is headed toward a shift from traditional chemical-based methods to a more ecofriendly alternative. In this context, biocatalysis is seen as a cost-effective, energy saving, and clean alternative. It is meant to catalyze degradation of recalcitrant chemicals in an easy, rapid, green, and sustainable manner. One already established application of biocatalysis is the removal of dyes from natural water bodies using enzymes, notably oxidoreductases like laccases, due to their wide range of substrate specificity. In order to boost their catalytic activity, various methods of enhancements have been pursued including immobilization of the enzyme on different support materials. Aside from increased catalysis, immobilized laccases have the advantages of higher stability, better durability against harsh environment conditions, longer half-lives, resistance against protease enzymes, and the ability to be recovered for reuse. This review briefly outlines the current methods used for detoxification and decolorization of dye effluents stressing on the importance of laccases as a revolutionary biocatalytic solution to this environmental problem. This work highlights the significance of laccase immobilization and also points out some of the challenges and opportunities of this technology.

## Introduction

Throughout the last century, several industries have inflicted a high demand for synthetic dyes. An estimate of 700,000 tons of different synthetic dyes are produced annually (Holkar et al., 2016; Bilal et al., 2017; Katheresan et al., 2018). Those dyes have stable chemical structures that make them resistant to degradation by heat, light, or water (Lu et al., 2012a, b; Singh and Gupta, 2020). Some even form water-soluble complexes that are toxic to human, animal, and marine life (Bilal et al., 2019). Moreover, hazardous chemicals used in the dye industry, like corrosive acids, hydrogen peroxide, and caustic soda, are abundantly found in dye wastewater (Katheresan et al., 2018). Major industries as leather and textile are guilty of discarding untreated used dyes in waste water. Other high dye-demanding fields, including electroplating, paper, pulp, tannery, plastic, pharmaceutical, and cosmetic industries (Husain, 2010), are also responsible of causing irreparable damage to the ecosystem through the way they release their wastes containing dyes into clean water bodies (Holkar et al., 2016; Katheresan et al., 2018; Shakerian et al., 2020).

The conventional chemical processes currently used are effective in dye degradation; nevertheless, they lead to the production of toxic intermediate products (Bilal et al., 2017, 2019; Rasheed et al., 2019). Consequently, utilizing technologies that are effective, cheap, and environmentally friendly are being highly favored by governments and dye manufacturers (Ashrafi et al., 2013; Sharma et al., 2018; Deska and Kończak, 2019; Shakerian et al., 2020). Accordingly, biocatalysis is considered a clean process of dye degradation. Various biocatalysts like oxidoreductases are used in degrading hazardous compounds, including phenolic pollutants, and natural or synthetic dye wastes (Bilal et al., 2018, 2019). Laccase is one widely used oxidoreductase catalyst of interest due to its catalysis potential of different dyes (Bilal et al., 2019). As a matter of fact, new sources for fungal and bacterial laccases have been increasingly demanded to degrade dye effluents, or most commonly known as dye waste (Dauda and Erkurt, 2020; Jeon and Park, 2020; Joshi et al., 2020; Singh and Gupta, 2020).

In the past decade, scientists have been working on the development of novel immobilization methods and support materials for laccases to improve their performance and reusability. This improvement is due to the noteworthy enhancement in pH and thermal stability profile range, as well as the capability of working under a wider range of environmental conditions (Dai et al., 2016; Ba and Vinoth Kumar, 2017; Skoronski et al., 2017; Ali et al., 2020).

The aim of this review is to highlight the recent and distinctive application of immobilized laccases in the degradation of dyes in waste water, and how enzyme immobilization enhanced its biocatalysis activity. Finally, limitations and future opportunities of using immobilized laccase are discussed.

## Recent Dye Removal Methods

Dyes are complex unsaturated organic molecules that are able to absorb light and give color, through reflecting the fraction of light not absorbed by the dye. They are categorized based on their chromophore structure, particle charge after dissolution, color index number, and industrial application as summarized in Supplementary Table 1 (Hunger, 2004; Yagub et al., 2014; Zhou et al., 2019; Benkhaya et al., 2020). Dyes and metal ions are the most prevalent detrimental materials found in dye wastewater that are very harmful to water and soil (Gosavi and Sharma, 2014; Holkar et al., 2016). Attention has been drawn lately toward remediation of dye wastewater for reuse, due to scarcity of clean natural water sources. An effective dye removal method ideally does its task rapidly, cost effectively, and without producing secondary contaminants (Rodríguez-Couto et al., 2009; Katheresan et al., 2018). The current established dye removal methods are classified into three main categories: physical, oxidation, and biological methods.

### Physical

The first physical dye removal method is the coagulation (flocculation) method. This method is mainly used for good removal of disperse dyes (Liang et al., 2014; Yeap et al., 2014), but it has the disadvantage of increased generation of sludge volume (Crini and Lichtfouse, 2019). Another physical method is adsorption, which has higher efficiency in discoloring more types of dyes than the coagulation method (Jadhav and Srivastava, 2013). It is also considered a cheap method of water remediation if low cost adsorbents, like polymeric resins and bentonite clay, are used, but this is not a cost-effective method as the adsorbent is usually used once, generating sludge, with no chance of regeneration for future use (Gupta et al., 2011). Third is the filtration method, where techniques like reverse osmosis and ultrafiltration are used to restore the effluent dye for commercial reuse, but the constant problems of these techniques are the high cost of filtration membranes and their maintenance (Holkar et al., 2016; Katheresan et al., 2018).

### Oxidation

This is an easily applied method for dye degradation; hence, it is the most commonly used, starting with advanced oxidation processes, where they can oxidize a wide range of chemicals, including organic and inorganic compounds found in wastewater, but it has the limitation of forming a precipitating sludge (Babuponnusami and Muthukumar, 2014). Then there are the chemical oxidation processes that are very powerful and can break down even the chemical structures of dyes with double bonds and complex aromatic rings using ozone (O_3_) molecules (Asghar et al., 2015). Nevertheless, using these molecules releases toxic secondary products as well as being expensive to purchase (Holkar et al., 2016). Finally, there are the synergistic hybrid advanced oxidation processes. It is a combination of the two previous methods, which is advised to be used when synergistic oxidative decolorization effect is desired (Holkar et al., 2016). However, it still has the disadvantages of the previous two methods.

### Biological

This method is the most preferred nowadays for degrading, detoxifying, and remediation of recalcitrant dyes from factory effluents. Its mechanism is based on the adaptability of the selected microorganisms and the strength of the biological enzymes either secreted directly from microorganisms or free enzymes (Solís et al., 2012). Preference of biological methods over physical and oxidation practices is attributed to their environmental benefits as there is no production of hazardous byproducts as well as less sludge formation. Moreover, it is a cost-effective method, which makes it a better candidate to be used in industrial scale (Hayat et al., 2015). Up to this point, many microorganisms and their enzymes have been studied and tested for their potential ability in degrading dyes found in wastewater (D’Souza-Ticlo et al., 2009; Wikee et al., 2019). Apart from the enzyme source, those enzymes degrade dyes through biocatalytic oxidation of their chromophores; hence, the increased interest in studying proprieties of different biocatalysts lately (Chapman et al., 2018). A number of research reports have documented the high efficiency of using enzymes in dye removal (Chiong et al., 2016; Yang X. et al., 2016; Katheresan et al., 2018; Kashefi et al., 2019b).

As promising as using enzymes commercially may seem, they still have their limitations like low stability and lack of recovery decreasing their potential for reuse. Also, rapid loss of catalytic activity may occur in case of altering their favorable operating conditions. Hence, they usually fail to perform under harsh industrial conditions. Therefore, new methods are being developed to enhance the durability of enzymes through immobilization. This is particularly crucial when intracellular enzymes are desired to be used in a cell free system. Nowadays, the usage of enzymes immobilized on solid carriers is gaining popularity more than the free enzyme, as immobilization stabilizes the protein structure giving it longer shelf-life, resistance against proteases, thermal and pH stability, and repeatability of use, thus, reducing operational cost, which makes a good candidate for commercial and industrial use (Dodor et al., 2004; Fernández-Fernández et al., 2013; Nair et al., 2013; Li et al., 2014; Sun et al., 2015; Bilal et al., 2019; Deska and Kończak, 2019).

## Immobilized Laccase in Removal of Dyes

One enzyme of interest that has been around for some time, is laccase. Scientists have regained interest in it due to its promising catalytic and physiochemical properties, making it the perfect candidate for bioremediation processes (Rao et al., 2014; Legerska et al., 2016). Laccase is a monomeric, dimeric, or a tetrameric glycoprotein, which oxidizes a broad spectrum of phenolic and non-phenolic substrates (Giardina et al., 2010). By now, many laccases have been discovered, and as a family, they show different structures and functions depending on their source as they can be widely found in eukaryotes as well as prokaryotes (Deska and Kończak, 2019); including higher plants, bacteria, insects, fungi, and recently mammals (Giardina et al., 2010; Janusz et al., 2020). As for the mechanism of action of laccases, they catalyze three types of reactions using oxygen atom and releasing water molecule: (i) direct oxidation of phenolic substrates, (ii) indirect oxidation of non-phenolic substrates with high redox potential in the presence of a natural or synthetic low molecular weight mediator (Breen and Singleton, 1999; Agrawal et al., 2018), and (iii) coupling reactions with reactive intermediate radicals formed during direct oxidation (Polak and Jarosz-Wilkolazka, 2012). The active site of laccases, where biocatalysis occurs, has three copper centers/types/domains; (1) blue copper center (type I), (2) normal copper center (type II), and (3) coupled binuclear copper centers (type 3) (Messerschmidt and Huber, 1990; Solomon et al., 1992; Dwivedi et al., 2011).

**FIGURE 1:**
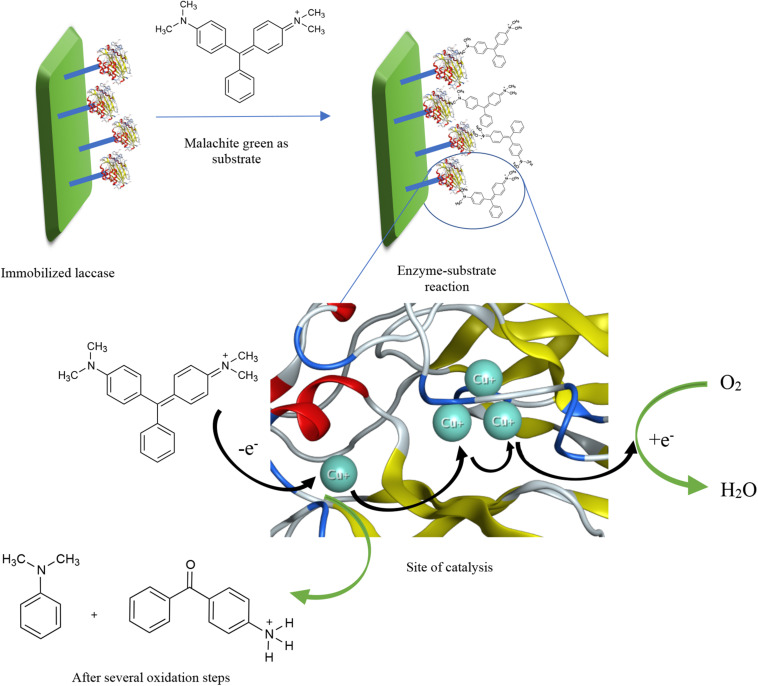
A proposed mechanism of dye degradation by immobilized laccase with the aid of the crystal structure of laccase from *Thermus thermophilus HB27* (PDB code: 5JRR).

A great number of research articles have reported the increased biocatalytic performance of laccase for a longer stretch of time when immobilized. As a general definition, to immobilize an enzyme is when the soluble form of the enzyme is attached to a solid support or, most recently, through formation of aggregates. In this context, different ways of immobilization include adsorption on beads or a matrix, covalent binding to a solid support, entrapment or encapsulation in polymers, and crosslinking as illustrated in Supplementary Figure 1 (Shrivastava et al., 2012; Datta et al., 2013; Sirisha et al., 2016). Whichever method is chosen, one point ought to be taken into consideration: no immobilization method should influence the enzymatic conformation so that the activity shall not be affected (Shaheen et al., 2017). Other factors to bear in mind while considering the different immobilization methods shall be more elaborated in Supplementary Data 1. A simple proposed mechanism of dye degradation by immobilized laccase is illustrated in Figure 1 with the aid of the crystal structure of laccase from *Thermus thermophilus HB27* (PDB code: 5JRR). In the figure, the dye structure, substrate, is being oxidized by only oxygen in the presence of laccase as a catalyst. When the dye is oxidized, it loses an electron that moves from one Cu^2+^ atom to another inside the catalytic site, until it eventually reduces oxygen to release water. More information about laccase-catalyzed descoloration of dyes and the exact oxidized products have been reported in literature (Zille et al., 2005; Yang et al., 2015; Kagalkar et al., 2015).

Immobilized laccases proved to be worthy challengers for the effective decolorization, degradation, and removal of dyes (Bilal et al., 2019). For example, laccase from the *Cerrena* sp. strain HYB07 was immobilized by preparing cross-linked enzyme aggregates (CLEAs) of the enzyme to degrade Remazol Brilliant Blue Reactive dye. Almost 90% of the dye was eliminated from the solution in just 40 min without the help of a redox mediator (Yang J. et al., 2016). In another study, Arica et al. (2017) demonstrated the immobilization of laccase on fibrous polymer-grafted polypropylene chloride film, where three dyes, namely, Procion Green H4G, Brilliant Blue G, and Crystal Violet, were tested for removal by both the free and immobilized enzyme. The immobilized laccase gave better dye degradation results than the free one (Arica et al., 2017).

In addition to the improved biocatalytic properties, immobilized laccase shows notable storage stability and repeated use capability, while maintaining satisfactory efficiency (Deska and Kończak, 2019). In a study, by Ma et al. (2018), genipin-activated chitosan beads were introduced as a highly stable laccase biocatalyst from *Trametes pubescens*. The resulting immobilized laccase reached to a decolorization of 77.49% of Acid Black 172 dye. Moreover, the carrier-supported laccase displayed excellent reuse potential with > 55% of remaining activity reserved after 11 cycles of constant use. It also showed high storage stability retaining over 57.14% of its original activity after 30 days of storage at 4°C (Ma et al., 2018).

Also, increased pH stability is a notable characteristic of the immobilized laccase as stated by Wen et al. (2019), when the enzyme was physically adsorbed on kaolinite for the removal of malachite green dye. The immobilized biocatalyst exhibited outstanding stability over a broad pH range from 3 to 6, with the lowest relative activity being 60% at pH 5.5 and the highest is 100% at pH 4.5. The authors justified this enhanced pH tolerance to a relative stable proton production on the surface of support and assured that it could retain the activity of laccase (Wen et al., 2019).

Furthermore, immobilized catalysts often show remarkable improved thermal steadiness than free enzymes. A recent report presented a novel support carrier; laccase covalently bound to crosslinked graphene oxide–zeolite nanocomposites. The obtained catalytic system was exploited for the degradation of Direct Red 23 dye. Notably, the immobilized laccase was very stable at 80°C and preserved 84% of its initial catalytic activity, unlike its free counterpart, which could only retain 18%. The author attributed this thermal protection to the covalent bonding between laccase and supporting carrier (Mahmoodi and Saffar-Dastgerdi, 2020).

Many literature reports confirm that using immobilized laccases is a favorable technology in the treatment of dye effluents. Several recent reports regarding this matter are listed in Table 1.

**TABLE 1:** Some recent reports about the degradation of dyes by immobilized laccases from different microbial sources.

Laccase source	Immobilization method	Immobilization matrix	Target molecule (dye)	Degradation (%)	References
White rot fungus *T. trogii*	Covalent binding	Thiolated chitosan–Fe_3_O_4_ hybrid composite	Reactive Blue 171, Acid Blue 74	Using 6 mg of immobilized NPs, 79% after 10 cycles and 56% after 8 cycles respectively	Ulu et al., 2020
*Trametes versicolor*	Adsorption Covalent binding	PMMA/PANI electrospun fibers	Remazol Brilliant Blue R	87% using adsorbed laccase and 58% using covalently bonded laccase	Jankowska et al., 2020
*Trametes versicolor*	Adsorption	Carbon nanotube nanocomposites	Congo Red	96% within 3 h	Zhang et al., 2020
N/A	Adsorption	3D PVA-co-PE HPNM for immobilizing laccase-Cu_2_(PO_4_)_3_⋅3H_2_O HNF.	Reactive Blue 2, Acid Blue 25, Acid Yellow 76, Indigo Carmine	83.59%, 86.35%, 90.2% in 10 h, 99.5% in 3 h respectively	Luo et al., 2020
N/A	Adsorption	CNT/GO&Lac@UF	Methylene Blue	80%	Zhu et al., 2020
*Trametes versicolor*	Adsorption	Poly(2-hydroxyethyl methacrylate-glycidyl methacrylate) [p(HEMA-GMA)] cryogels	Brilliant Blue R, Brilliant Green, Orange G, Procion Red MX-5B, Congo Red, Sunset Yellow	63.16%, 77.27%, 52.27%, 34.71%, 46.67%, 52.08% respectively	Bayraktaroğlu et al., 2020
*Trametes versicolor*	Entrapment	PILM	Remazol Brillant Blue R	75%	HajKacem et al., 2020
*Boletus edulis*	Adsorption	Modified rice husks	Reactive Blue-19	91%	Tuncay and Yagar, 2020
N/A	Adsorption	TiO_2_ sol–gel-coated PAN/O-MMT composite nanofibers	Crystal Violet	95% in 6 h	Wang et al., 2020
Genetically modified *Aspergillus*	Covalent attachment	Graphene oxide nano-sheets	Direct Red 23, Acid Blue 92	88.7%, 48.7% respectively	Kashefi et al., 2019a
*Trametes versicolor*	Cross-linking	Laccase-Cu_3_(PO_4_)_2_⋅3H_2_O hybrid NFs	Bromophenol Blue, CBBR-250, Xylene cyanol	41.2%, 73.2%, 73.0% respectively (without use of mediator)	Patel et al., 2018
*Myceliophthora thermophila*	Covalent attachment	Epoxy-functionalized silica	Malachite Green, Acid Red 52, Acid Orange 156, Coomassie Brilliant Blue, Methyl Violet	100%, 99%, 98%, 97%, 78% respectively; all in the presence of DMHBA as redox mediator	Salami et al., 2018
*Trametes versicolor*	Adsorption	Methacrylyol group and amino ] group functionalized Fe_3_O_4_@SiO_2_	Methyl Red	Removal efficiency was more than 80% for the first 3 days	Lin et al., 2017
*Trametes pubescens*	Entrapment	Chitosan beads	Blue R, Indigo Blue, Remazol Brilliant, Reactive Brilliant, Blue X-B, Methylene Blue, Acid Black 172, Neutral red, Congo Red, Naphthol Green B	68.84%, 56.28%, 54.24%, 52.26%, 48.23%, 45.12%, 44.58%, 37.18%, 25.39%, 20.81% respectively	Zheng et al., 2016
*Trametes versicolor*	Encapsulation	Sponge-like chitosan grafted polyacrylamide hydrogel	Malachite Green	90% in the first cycle	Sun et al., 2015

*DMHBA, 3,5-dimethoxy-4-hydroxybenzaldehyde; PMMA/PANI, poly(methyl methacrylate)/polyaniline; HNF, hybrid nanoflower; HPNM, hierarchically porous nanofibrous membrane; CNT/GO&Lac@UF, carbon nanotubes/graphene oxide and laccase on ultrafiltration membrane; PILM, polymeric ionic liquid membrane; PAN/O-MMT, polyacrylonitrile/organically modified montmorillonite; ABTS, 2,2’-azino-bis (3-ethylbenzothiazoline-6-sulfonic acid); NFs, nanoflowers.*

## Limitations and Future Opportunities

As narrated throughout this minireview, the current established dye treatment methodologies still have, to some extent, several limitations. The fact that no certain decolorization method is adopted universally for all types of dye effluents needs more study, as currently, using a single dye removal method, either physical, oxidation, or biological, is not enough. Using a combination of different methods may be attributed to the complex and different chromophore structures of dying compounds. It is undeniable that the current enzyme immobilization methods are very useful and somehow effective in the issue at hand: decolorization and detoxification of dye wastewater. However, as auspicious as immobilized enzymes seem to be, the matter of loss of activity or stability should be addressed. Limitations of current immobilization methods are due to enzyme leakage from carrier, undesired reactions between carrier and enzyme or even the inability of the enzyme to react with its substrate. Nowadays, scientists are still developing new enzyme immobilization methods as it is a promising field that gives both better results and cost effectiveness for industrial use. However, from what is evident from recent research reports, they are merely focused on finding new materials, but the principles and methodologies of enzyme immobilization are still unchanged.

Despite the success of most of the reported laccase immobilization methods in dye removal, there is still one more aspect to be investigated when assessing their efficiency. The chemical structure of the target dye affects the results dramatically. Research reports often published assess the efficiency of their technologies by testing them on dyes with simple, low molecular weight structures that eventually show high decolorization results. Also, those promising technologies are still not implemented on an industrial scale. As the industrial sectors always seek the most economical technologies, they prefer using large quantities of mixed crude cultures or pure cultures over the immobilized one to limit the costs, overlooking the long-term advantages of reusage of immobilized enzymes. Most are drawn toward fungal more than bacterial laccase, due to its higher redox potential that show a higher oxidative ability. However, bacterial laccases are reported to withstand the harsh industrial environments with higher temperatures and pH values than those tolerated by fungal laccases. It is advised to use a redox mediator to act as an electron shuttle for better decolorization results of dye structures with higher redox potentials.

For all the mentioned drawbacks, immobilized laccases represent a potential effective, eco-friendly and commercial alternative to the physical, chemical, and oxidative dye decolorization methods. The scientific and industrial communities have real chances and future opportunities to moderate the current situation. Improvements can be made in fields of developing novel immobilization strategies in order to avoid enzyme leakage and come up with a more stable, durable, sustainable, and economical immobilization systems. One solution to the problem is the immobilization of both enzyme and redox mediator on the same carrier. This method ensures that low and medium redox potential laccases have better efficiency and operational stability. Also, this co-immobilized system can be reused several times, which will eventually reduce the manufacturing cost, that is ultimately the main concern of the industrial sector. This ambitious suggestion shall introduce an environmentally conscious and cost-effective dye detoxification method to the market.

## Author Contributions

SAGZM: conceptualization, original draft preparation, and editing. AAT: manuscript reviewing. MSMA: supervision. FMS: project administration and supervision. All authors contributed to the article and approved the submitted version.

## Conflict of Interest

The authors declare that the research was conducted in the absence of any commercial or financial relationships that could be construed as a potential conflict of interest.

## Supplementary Material

Supplementary Figure 1 Basic methods of enzyme immobilization.

Supplementary Table 1 Classification of dyes according to industrial application, particle charge after dissolution and chromophore structure.

Supplementary Data 1 Types of immobilization methods.
