# Supplementary figures and images for: Current Development in Decolorization of Synthetic Dyes by Immobilized Laccases
Source: Front Microbiol. 2020 Sep 30;11:572309. doi: 10.3389/fmicb.2020.572309 (PMC7554347; doi:10.3389/fmicb.2020.572309)

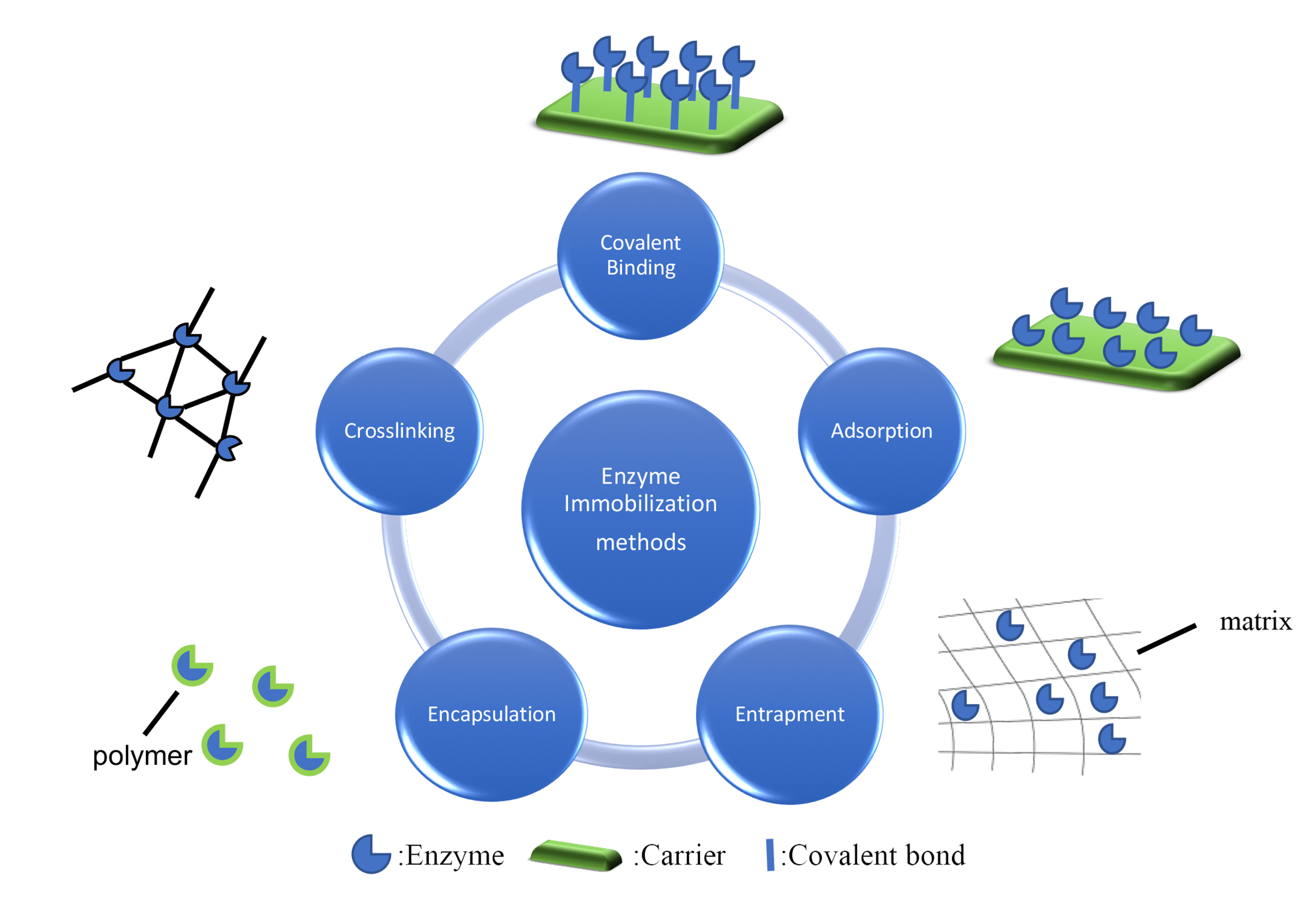

Supplement: Supplementary Figure 1 — Basic methods of enzyme immobilization. [file Image_1.TIF]
