# Supplementary material for: Current Development in Decolorization of Synthetic Dyes by Immobilized Laccases
Source: Front Microbiol. 2020 Sep 30;11:572309. doi: 10.3389/fmicb.2020.572309 (PMC7554347; doi:10.3389/fmicb.2020.572309)
Supplement: Supplementary Table 1 — Classification of dyes according to industrial application, particle charge after dissolution and chromophore structure. [file Table_1.DOCX]

Supplementary Material

**Table ST1. Classification of dyes according to industrial application, particle charge after dissolution and chromophore structure.**

| Type | Chemical class/ chromophore structure | Water solubility | Substrate | Example |
| --- | --- | --- | --- | --- |
| Disperse dye  (non-ionic) | Azo, nitro, styryl, anthraquinone, benzodifuranone, quinonaphthalone, arylbenzimidazole, aminonaphthlimide, naphthoquinoneimine | Insoluble or sparingly soluble | Polyester, nylon, cellulose, cellulose acetate, acrylic fibers, polyamide, plastics | 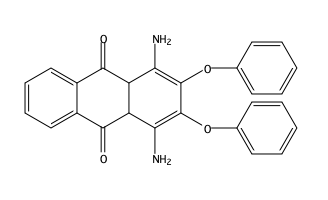 Disperse violet 26 |
| Direct dye  (anionic) | Azo, oxazine, thiazole, stibene, phthalocyanine | soluble | Paper, cellulose fibers, nylon, rayon, cotton, viscose, leather | 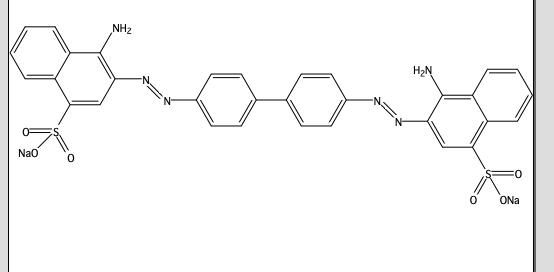 Direct red 28 |
| Reactive dye  (anionic) | Anthraquinone, formazan, oxazine, phthalocyanine, azo | soluble | Cellulose fibers, silk, cotton, wool fibers, nylon | 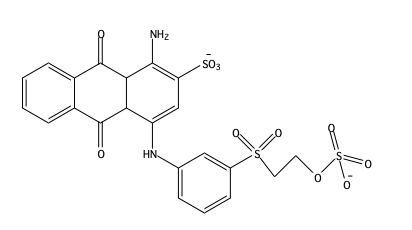 Reactive blue 19 |
| Vat dye  (non-ionic) | Anthraquinone, Indigoid | Insoluble and  some are soluble in presence of Na_2_CO_3_ | Cellulose fibers, cotton, viscose, wool | 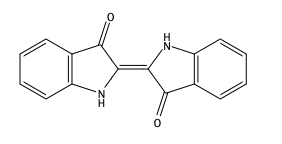 vat blue 1 |
| Basic dye  (cationic) | Triarylmethane, azo, xanthene, Triphenylmethane, hemicyanine, cyanine, acridine, diazahemicyanine, anthraquinone, oxazine, thiazine | soluble | Acrylic, ink, paper, silk, wool, cotton, treated nylon, modified polyester, polyacrylonitrile | 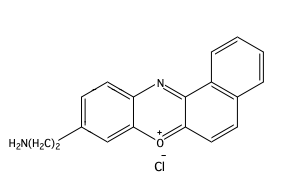 Basic blue 6 |
| Acid dye  (anionic) | Anthraquinone, xanthene, azo, nitrodiphenylamine, triphenylmethane, nitroso, azine, nitro, indigoid, quinoline and carbolan | soluble | Nylon, wool, leather, food, silk, cotton, cosmetics, ink-jet printing, paper, modified acrylics | 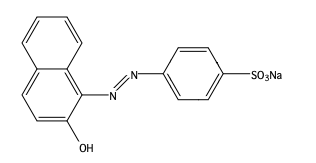 Acid orange 7 |
