# Supplementary material for: Current Development in Decolorization of Synthetic Dyes by Immobilized Laccases
Source: Front Microbiol. 2020 Sep 30;11:572309. doi: 10.3389/fmicb.2020.572309 (PMC7554347; doi:10.3389/fmicb.2020.572309)
Supplement: Supplementary Data 1 — Types of immobilization methods. [file Data_Sheet_1.PDF]

Supplementary Material

Supplementary data 1. Types of immobilization methods

Covalent binding

This type is considered tedious based on the fact that a chemical linkage is made between the enzyme and the support, which requires extra pretreatment. Firstly, a specific group on the support is chemically activated. Then, and this is the harder part, this activated group irreversibly binds to a functional group from the enzyme that is not essential for catalysis to occur. This method provides stronger binding (covalent bond) between the enzyme and the support, insuring that the enzyme is not leaked during the desired application. It also significantly stabilizes the tertiary protein structure making it more resistant to different temperature, pH and solvents, presenting high enzymatic activity (Shakerian et al., 2020).

At the first glance, this method seems satisfactory but it has its cons including the high processing cost and the impossibility of regenerating the used solid carrier for a different use. And above all, there is the possibility of altering the structure of the active site of the catalyst (Rao et al., 2014).

Adsorption

Unlike covalent binding, enzyme adsorption is simple, fast and does not require chemical pretreatment of the solid support. The enzyme is bound to the solid support by weaker bonds like Van der Waal's, hydrogen bonds and ionic interactions. The activity of adsorbed enzymes is fairly acceptable. Additionally, adsorption has numerous advantages like its low processing cost and the ability of regenerating the used carrier. However, some enzyme leakage may occur due to medium strength bonds as well as the low stability of the catalytic system (Jesionowski et al., 2014).

Entrapment

An enzyme is entrapped within tiny spaces of an organic or inorganic synthetic matrix. In order to do so, usually the supporting fiber is fabricated in the presence of the desired enzyme (Sheldon & van Pelt, 2013). This method is quite controversial when it comes to assessing its pros and cons. For instance, the enzyme gains great features when immobilized as increased stability, yet it is continuously leaked due to varying pore sizes of the carrying fiber and medium strength bonds. This method allows minimum alterations to the enzyme structure, but still the resulting enzymatic activity is low because of reduced substrate accessibility to the enzyme as a result of steric hindrance. In the same context, low quantity of enzyme is needed for immobilization, yet the immobilization process itself is difficult and costly. Finally, the used fibers provide resistance against solvents, microbes and pH, nevertheless it is impossible to regenerate the matrix again (Rao et al., 2014).

Encapsulation

The enzyme molecule is surrounded by a semipermeable membrane that allows the substrate in, but does not allow the enzyme out. Not only does this method provide a large surface area for the enzyme

and the substrate to react together giving the change for a high enzymatic activity, but also several enzymes can be immobilized at the same time. More advantages include the possibility of regenerating the support which also provides protection against microbial attacks. Still, this costly immobilization technique is guilty of requiring a very high enzyme concentration (Sheldon & van Pelt, 2013; Bilal et al., 2017).

Cross-linking

Several macromolecules of the same enzyme are joined together through small bifunctional molecules, all aggregated together in the same spot. As a pro, the crosslinked enzyme aggregates formed are resistant to extreme temperature and pH conditions, highly stable and can withstand microbial attacks. On the contrary, the enzyme activity is low or may even be lost as the active site of the enzyme may have a role in the immobilization due to difficulty in controlling the chemical bonding reaction. Also, the need for high concentration of the enzyme to begin with is another drawback (Sheldon & van Pelt, 2013; Sharma et al., 2018; Deska & Kończak, 2019).

Bibliography:

Bilal, M., Asgher, M., Parra-Saldivar, R., Hu, H., Wang, W., Zhang, X., & Iqbal, H. M. N. (2017). Immobilized ligninolytic enzymes: An innovative and environmental responsive technology to tackle dye-based industrial pollutants – A review. *Science of the Total Environment*, *576*(1), 646–659. https://doi.org/10.1016/j.scitotenv.2016.10.137

Deska, M., & Kończak, B. (2019). Immobilized fungal laccase as “green catalyst” for the decolourization process – State of the art. *Process Biochemistry*, *84*(1), 112–123. https://doi.org/10.1016/j.procbio.2019.05.024

Jesionowski, T., Zdarta, J., & Krajewska, B. (2014). Enzyme immobilization by adsorption: A review. *Adsorption*, *20*(5–6), 801–821. https://doi.org/10.1007/s10450-014-9623-y

Rao, M. A., Scelza, R., Acevedo, F., Diez, M. C., & Gianfreda, L. (2014). Enzymes as useful tools for environmental purposes. *Chemosphere*, *107*(1), 145–162. https://doi.org/10.1016/j.chemosphere.2013.12.059

Shakerian, F., Zhao, J., & Li, S. P. (2020). Recent development in the application of immobilized oxidative enzymes for bioremediation of hazardous micropollutants – A review. *Chemosphere*, *239*(1), 1–12. https://doi.org/10.1016/j.chemosphere.2019.124716

Sharma, B., Dangi, A. K., & Shukla, P. (2018). Contemporary enzyme based technologies for bioremediation: A review. *Journal of Environmental Management*, *210*(1), 10–22. https://doi.org/10.1016/j.jenvman.2017.12.075

Sheldon, R. A., & van Pelt, S. (2013). Enzyme immobilisation in biocatalysis: Why, what and how. *Chemical Society Reviews*, *42*(15), 6223–6235. https://doi.org/10.1039/c3cs60075k
